# Structural and Functional Impact of Spinal Cord Stimulation in Diabetic Peripheral Neuropathy

**DOI:** 10.7759/cureus.103728

**Published:** 2026-02-16

**Authors:** Mustafa Almosawi, Rofyontsa Shanti, Norah Hill, Kelly Gartner, Bryce Ahn, Rodrigo Fernández-Gajardo, Nelleke van Wouwe, Michael Pulla, Timothy Ford, Nicholas Ahn, Ajmal Zemmar

**Affiliations:** 1 Neurological Surgery, University of Louisville School of Medicine, Louisville, USA; 2 Orthopedic Surgery, University of Louisville School of Medicine, Louisville, USA; 3 Neurological Surgery, University of Louisville Hospital, Louisville, USA; 4 Podiatry, Robley Rex Veterans Affairs (VA) Medical Center, Louisville, USA; 5 Orthopedics, University of Louisville School of Medicine, Louisville, USA; 6 Orthopedics, University of Louisville Hospital, Louisville, USA; 7 Neurological Surgery, Robley Rex Veterans Affairs (VA) Medical Center, Louisville, USA

**Keywords:** diabetic peripheral neuropathy, dorsal root ganglion, dorsal root ganglion stimulation (drgs), nerve conduction study (ncs), nerve regeneration, neuromodulation, neuroplasticity, painful diabetic neuropathy, small fiber neuropathy, spinal cord stimulation (scs)

## Abstract

Diabetic peripheral neuropathy (DPN) is a common and debilitating complication of diabetes that leads to chronic pain, sensory loss, and functional decline. While spinal cord stimulation (SCS) is FDA-approved for painful diabetic neuropathy, its potential to modify disease pathology rather than merely suppress symptoms remains poorly understood. This narrative review bridges this knowledge gap by elucidating the role of SCS in mitigating structural and functional changes in the spinal cord and the peripheral nervous system in DPN. A systematic PubMed search for studies published between January 1990 and May 2025 on the structural, functional, vascular, or biochemical effects of SCS in diabetic neuropathy yielded 361 records, of which 22 met the inclusion criteria. Data were extracted thematically across five domains: peripheral nerve structure, spinal plasticity, supraspinal modulation, neuroinflammation, and vascular/metabolic effects. SCS consistently improved both functional and structural biomarkers of neuropathy. In clinical studies, SCS enhanced nerve conduction velocity, increased intraepidermal nerve fiber density, and promoted corneal nerve regeneration within 6-12 months. Preclinical data demonstrated suppression of microglial activation, downregulation of pro-inflammatory mediators, and restoration of neurotrophic signaling. SCS also improved spinal and peripheral microcirculation and reversed metabolic and vascular dysregulation associated with hyperglycemia. Collectively, these effects suggest that SCS has multimodal benefits that can restore neural integrity, recalibrate neuroimmune pathways, and mitigate disease progression in DPN. These findings position SCS as a potential disease-modifying therapy for DPN and underscore the need for prospective mechanistic trials integrating structural and functional biomarkers to refine patient selection and optimize neuromodulation outcomes.

## Introduction and background

Diabetic peripheral neuropathy (DPN) is a debilitating complication of diabetes mellitus that develops in half of patients with the disease [1,2]. With diabetes projected to affect one-third of the global population by 2050, the clinical and socioeconomic burden of diabetic neuropathy is expected to be staggering [2]. Over time, chronically high blood glucose levels damage peripheral nerves, leading to pain, numbness, and tingling, particularly in the feet and legs. As nerve damage progresses, patients may experience muscle weakness, an unsteady gait, and diminished quality of life [3,4]. Falls are an even greater clinical challenge, as individuals with DPN are up to 20-fold more likely to fall compared to age-matched non-diabetic controls [5]. For 30-50% of those with DPN, the condition takes an even more severe form, painful diabetic neuropathy (PDN) [1,2]. Genetic factors, metabolic disturbances, lifestyle, and environmental triggers all influence the progression of DPN to painful DN [2,6]. Typically, pain is most prevalent in the extremities because the longest nerves in the body are most vulnerable to damage, and approximately 80% of patients with PDN report severe foot pain [7]. Left unchecked, continuous nerve injury not only contributes to painful sensations but also increases the risk of diabetic foot ulcers [8]. Compounding the problem, diabetes also undermines overall vascular health, setting the stage for diabetic foot syndrome and, in the worst cases, major limb amputation, which accounts for more than 80% of medically indicated amputations [9]. Nontraumatic, major, lower extremity amputation has high risks of mortality, with pooled rates of 27.3% at one year and 63.2% at five years among diabetic patients [10].

Though the effects of DPN are many, the therapeutic arsenal remains modest. Initially, management of DPN and PDN centers on tight blood sugar control alongside first-line agents like pregabalin, gabapentin, and serotonin-norepinephrine reuptake inhibitors [11,12], as well as non-pharmacological measures. For drug-refractory pain, spinal cord stimulation (SCS) has emerged as a compelling treatment option. Clinical studies report that SCS can not only deliver pain relief and improve quality of life but also help patients lose excess weight and achieve better glycemic control, likely because increased mobility due to pain relief facilitates weight loss. These benefits appear to be most pronounced among individuals with higher baseline body mass index (BMI) and hemoglobin A1c (blood glucose) levels [13-16]. Recent expansions in FDA approvals have significantly widened the eligibility for SCS implants in diabetic neuropathy [15], and early clinical data confirm that these devices can be safely placed even in patients with elevated hemoglobin A1c without increased complication rates [17]. Additionally, alternative neuromodulation modalities, such as dorsal root ganglion stimulation (DRGS) and peripheral nerve stimulation (PNS), have accumulated evidence supporting their efficacy in improving sensory perception and functional mobility in diabetic neuropathy [18-20]. However, the mechanisms behind these effects of neuromodulation in the treatment of DPN remain unclear.

This narrative review bridges the knowledge gap regarding the effects of SCS on DPN by reviewing its role in mitigating structural and functional changes in the spinal cord and the peripheral nervous system. In the following sections, we outline the principal structural and functional biomarkers used to monitor the progression of peripheral neuropathy and examine how SCS and other adjunctive neuromodulation techniques influence pathological changes in the nervous system and modulate specific biomarkers. This study may inform healthcare professionals about the effectiveness of SCS for DPN and provide valuable insights to refine patient selection algorithms and improve overall therapeutic outcomes.

## Review

Pathogenesis of PDN

A quarter of patients with diabetes develop peripheral nerve damage within five years of diagnosis, with rates rising above 40% by the 10-year mark [3]. Central to this process is oxidative stress from chronic hyperglycemia (i.e., high glucose levels). High glucose levels in patients with diabetes drive overproduction of reactive oxygen species, which injure neurons directly and impair the microvasculature (vasa nervorum) that delivers oxygen and nutrients to peripheral nerves, thereby further compromising nerve survival and function [21,22]. Persistent hyperglycemia also initiates a cascade of metabolic, neural, inflammatory, and vascular alterations, resulting in axonal degeneration, microglial activation, and impaired nerve conduction [23,22]. Interestingly, transcriptomic profiling of the dorsal root ganglia in patients with PDN reveals a surge in inflammation-related gene expression, particularly from infiltrating macrophages, driving heightened pain sensitivity [24]. Metabolic dysfunctions may further exacerbate PDN pathophysiology, as an enzyme-driven switch toward glycolysis in the dorsal root ganglion (DRG) appears to drive neuroinflammation and increase central pain signaling [25]. Clinically, this damage presents as the classic “glove and stocking” pattern, in which the longest peripheral nerves, those reaching the feet and hands, are affected first [2]. Together, these intertwined metabolic, inflammatory, and vascular insults set the stage for the progressive, length-dependent neuropathy so common in diabetes.

Structural and functional changes in the CNS in DPN

The impact of diabetes on the nervous system extends far beyond the periphery. DPN is associated with numerous structural and functional alterations in the DRG, spinal cord, and supraspinal pain networks, thereby driving chronic neuropathic pain through mechanisms such as central sensitization and inflammation.

Magnetic resonance imaging (MRI) has revealed that individuals with DPN often exhibit structural changes in the spinal cord, most notably, a reduction in cervical cross-sectional area [26,27]. Parallel studies in type 2 diabetic animals echo these findings, showing spinal atrophy alongside markers of inflammation and an unexpected increase in oligodendrocyte numbers, even when overall cord size remains stable [28]. Models of PDN demonstrate increased myelinated fibers in the spinothalamic tract and expanded oligodendrocyte populations in the dorsal horn, changes that amplify central sensitization and pain signaling [29]. Compounding this, enhanced synaptic density in the dorsal horn reflects maladaptive plasticity, rewiring the cord to be more responsive to pain inputs [30]. Together, these observations underscore how chronic high blood sugar and ongoing peripheral nerve injury can reshape spinal circuitry in ways that perpetuate neuropathic pain.

At the heart of this maladaptive spinal response lies the activation of microglia, the resident immune cells of the central nervous system (CNS) [31]. In the context of chronic hyperglycemia, oxidative stress provokes these cells to shift into a pro-inflammatory state, fueling central sensitization and the persistent pain of PDN [31]. Blocking microglial activation in animal models has been shown to alleviate neuropathic pain, underscoring the significant role of microglia in symptom perpetuation [32]. Upon activation, microglia release a cascade of cytokines and neuroactive mediators that amplify pain pathways and perpetuate the inflammatory cycle [31]. Targeting microglial signaling and inflammatory pathways may be a promising avenue to break this vicious cycle of inflammation and restore normal pain processing in diabetic neuropathy.

The DRG serves as a metabolic and immune “hotspot” within the PDN. In diabetes, the DRG shifts toward glycolysis, driven in part by upregulation of pyruvate dehydrogenase kinases (PDK2 and PDK4), which heightens neuronal excitability and pain sensitivity [25]. At the same time, infiltrating macrophages and an upsurge of pro-inflammatory gene expression promote local inflammation and even neuronal loss, further sensitizing peripheral pain fibers and giving rise to the burning, stabbing sensations that patients describe. Moreover, studies in diabetic mice showed that the activation of the NR2A-Wnt-TLR2 (NMDA receptor subunit; Wnt signaling pathway; toll-like receptor) signaling axis in the DRG contributes to sensitization by activating the microglia [33].

These maladaptive changes in the DRG extend upstream to the spinal dorsal horn, where they reinforce the hyperexcitability of glutamatergic neurons. PDN amplifies synaptic input and activity of excitatory amino acid receptors, including NMDA, AMPA, and mGluR5 receptors [34,35]. This is reflected by greater amplitude and frequency of excitatory postsynaptic currents in lamina II neurons, upregulation of calcium-permeable AMPA receptors, and enhanced phosphorylation of NMDA receptor subunits in diabetic models, all of which drive central sensitization and pain hypersensitivity [34,35].

Chronic high glucose levels also take a toll on the blood-spinal cord barrier. In PDN, hypoxia-driven changes in the microvasculature lead to endothelial cell loss and breakdown of this critical barrier, particularly within the dorsal horn [21]. Diabetes appears to blunt vascular endothelial growth factor-A (VEGF-A) signaling, thereby eroding capillary integrity and depriving dorsal horn neurons of oxygen and nutrients [21,36]. The resulting hypoxic stress further sensitizes these neurons, amplifying pain signaling [36].

At the same time, hyperglycemia-driven metabolic disturbances at the cellular level exacerbate structural and functional nerve injury. Hyperglycemia activates the polyol pathway, leading to sorbitol accumulation, myo-inositol depletion, and impaired Na⁺/K⁺-ATPase activity, thereby disrupting axonal electrophysiology and slowing nerve conduction velocity [37]. Additionally, advanced glycation end products, oxidative stress, and mitochondrial dysfunction further damage axons and Schwann cells, resulting in progressive loss of both small and large nerve fibers. The loss of intraepidermal nerve fibers (IENFs), the hallmark of small fiber neuropathy, correlates with the duration of diabetes and is detectable by skin biopsy. In contrast, nerve conduction studies reveal reduced conduction velocities and amplitudes as large fiber involvement progresses [38]. These structural and functional changes occur in parallel, and their severity correlates with the extent and chronicity of metabolic derangement, as demonstrated by histopathological and neurophysiological studies in patients with diabetes [37,38].

PDN disrupts the function of key supraspinal hubs that gate pain signals, such as the thalamus, hypothalamus, and brainstem. For instance, reduced connectivity of the ventrolateral periaqueductal gray, an area that normally modulates pain, is associated with greater pain intensity in patients [39]. At the same time, altered pathways connecting the thalamus, hypothalamus, and limbic system appear to underlie both the sensory and autonomic symptoms of diabetic neuropathy [40]. Structural MRI studies reveal that the cortex is not spared; both patients with painful and painless DPN exhibit thinning of the primary somatosensory and motor cortices, with more severe neuropathy correlating with greater somatomotor thinning [41]. Likewise, the ventrobasal thalamic nuclei shrink in all DPN patients. However, those with painful “non-irritable” nerve phenotypes show additional volume loss in somatosensory, posterior cingulate, and thalamic regions compared to their “irritable” counterparts.

Standard treatment approaches for PDN

Conservative management remains the cornerstone of care for PDN. Tight glycemic control, particularly in type 1 diabetes, can slow or even partially reverse nerve injury, as evidenced by lower rates of distal symmetric polyradiculoneuropathy among patients who maintain intensive glycemic targets [11,12]. However, in type 2 diabetes, the benefits of lowering blood-sugar levels are less consistent and must be balanced against the risk of hypoglycemia. Other non-drug interventions, including tailored physical therapy, therapeutic massage, aerobic exercise, and weight-bearing exercise, have been shown to improve sensory and motor performance, enhance postural stability, and improve overall quality of life [11,12]. When pain persists despite these measures, the first pharmacologic line includes anticonvulsants and serotonin-norepinephrine reuptake inhibitors, with careful dose titration to minimize side effects [11,12]. Additionally, tapentadol or tricyclic antidepressants may be considered for PDN, though safety concerns and tolerability often limit their use. Opioids should be avoided in PDN due to their modest analgesic gain and high-risk profile, underscoring the need for additional therapies that effectively relieve pain without compromising patient safety.

Neuromodulation techniques for PDN

Although conservative management remains the cornerstone of care for PDN, neuromodulation may be considered for patients with refractory PDN. Neuromodulation techniques target the ascending pain pathways at various levels, including the peripheral nerves, DRG, and dorsal columns of the spinal cord. These interventions can be categorized into tonic and burst SCS, closed-loop SCS, and target-based neuromodulation approaches, including DRGS and PNS [11,12,41]. These are clinically approved, fully implantable neuromodulation systems. Traditional tonic SCS operates at a frequency above the perception threshold and has shown modest to moderate efficacy in treating PDN [12]. Modern subthreshold stimulation methods, such as 10 kHz burst SCS, do not produce perceptible sensations but show significant pain control and high responder rates [14]. Randomized clinical trials have found that 10 kHz burst SCS provides substantial improvements in pain relief and overall health-related quality of life compared with conventional medical management alone, with sustained benefits for up to 12 months [14]. Patients treated with 10-kHz SCS plus conventional medical management had an 85% response rate with at least 50% pain relief at six months, a 76.3% reduction in lower limb pain (60% achieved sustained pain remission; VAS ≤3 cm), a 61.9% reduction in sleep disturbance, and a 17.7-point mean improvement in Global Assessment of Functioning [14]. These results have established SCS as a reliable therapy for pain management in PDN.

DRGS has shown comparable therapeutic efficacy to SCS in treating PDN at 6 and 12 months; approximately 80% of patients in each group achieved ≥50% reduction in pain intensity, with no statistically significant difference between treatments [18]. Both SCS and DRGS also led to significant improvements in health-related quality of life, as measured by EQ-5D and EQ-VAS scores, sustained throughout the study period. Notably, DRGS delivered clinical outcomes comparable to those of SCS, demonstrating its potential as an equally effective yet more targeted therapy for managing PDN [18]. DRGS may be preferable to SCS when pain is localized to discrete dermatomes, such as the distal feet, because it provides highly focal, targeted coverage of painful areas with minimal extraneous stimulation [18,42].

A promising advancement in this field is closed-loop stimulation, which uses real-time physiological feedback to adjust stimulation parameters on a pulse-by-pulse basis. Closed-loop SCS is a recent advancement that uses real-time measurement of evoked compound action potentials to dynamically adjust stimulation parameters, ensuring consistent neural activation and optimizing analgesic efficacy. It can automatically adapt to physiological changes, such as posture or movement, and maintain steady engagement of the dorsal column [41]. Given that PDN arises from aberrant sensory signaling, this precise and adaptive control offers a strong mechanistic rationale for its potential application in PDN, where maintaining stable modulation of dorsal column fibers could enhance both pain relief and treatment consistency [12,41].

PNS is gaining traction as a less invasive alternative for patients with refractory PDN, particularly when pain arises from a localized area. Recent clinical studies have demonstrated that stimulation of the posterior tibial nerve can significantly reduce foot pain in patients with neuropathic pain [19]. This dynamic approach helps maintain consistent neural activation within a therapeutic window, enhancing clinical efficacy despite patient movement or physiological fluctuations [43]. Closed-loop tibial nerve PNS has been shown to modulate wide-dynamic-range (WDR) neurons in the spinal dorsal horn, neurons commonly involved in chronic pain processing. In preclinical models, closed-loop PNS has been shown to normalize WDR firing patterns, suggesting that it may suppress pathological pain while preserving protective nociceptive signaling in PDN [44]. In parallel, closed-loop SCS has demonstrated significant benefits in other chronic pain populations, including improved pain relief, physical function, emotional well-being, and sleep quality [41]. However, the potential of these closed-loop systems to reverse the underlying pathophysiology of PDN and deliver sustained therapeutic benefits remains to be fully investigated. Moving forward, rigorously designed clinical trials will be essential to determine whether these adaptive stimulation systems can offer durable relief and functional restoration for patients with diabetic neuropathy. Our narrative review represents a fundamental step toward elucidating the direct effects of neuromodulation on CNS biomarkers in patients with diabetic neuropathy, focusing on peripheral nerve structure, spinal cord plasticity, supraspinal effects, neuroinflammation, and vascular/metabolic biomarkers. This study may inform healthcare professionals about the effectiveness of neuromodulation treatment for drug-refractory DPN and provide valuable insights to enhance overall therapeutic success.

Methods

Our narrative review has thus far discussed the pathophysiology of DPN, including structural and functional changes, and typical treatment options. To further elucidate the effects of neuromodulation on the CNS in DPN, we conducted a systematic PubMed search of articles published between January 1990 and May 2025. This review is supported by a systematic search and guided by core PRISMA 2020 reporting principles to promote transparency in search documentation, eligibility decisions, and data synthesis. The search strategy incorporated the following keywords in various combinations: [“diabetic peripheral neuropathy” OR “DPN”] AND [“spinal cord stimulation,” OR “SCS”]. Additional free-text searches were performed, and the reference lists of all included studies were screened to ensure comprehensive coverage of relevant publications.

The eligibility criteria included peer-reviewed human clinical trials or preclinical (animal) studies evaluating SCS in DPN that reported structural, functional, vascular, or biochemical outcomes. Studies were excluded if they were reviews, editorials, conference abstracts, or letters lacking original data, or if they focused on conditions unrelated to DPN or used unrelated stimulation modalities.

To extract and synthesize data, all identified citations were imported into Excel for initial de-duplication and screening. Three independent reviewers screened titles and abstracts. Full texts of potentially eligible studies were reviewed against the inclusion criteria. Any discrepancies were resolved by consensus. The reference lists of extracted studies were reviewed to identify additional relevant studies. Extracted variables included study design, sample size, population characteristics, SCS modality (e.g., tonic, 10 kHz, burst), intervention details, and key outcome metrics (pain scores, nerve fiber density, nerve conduction velocity, corneal biomarkers, microvascular flow, and imaging findings). Extracted data were categorized thematically across five domains: peripheral nerve structure, spinal cord plasticity, supraspinal effects, neuroinflammation, and vascular/metabolic biomarkers.

Results

**Figure 1:**
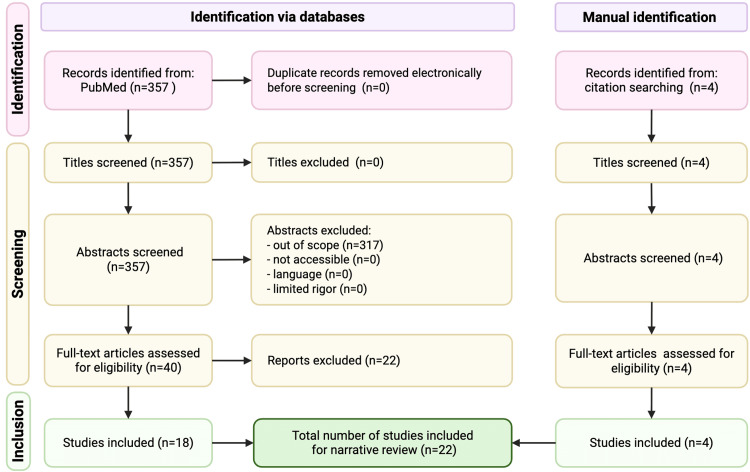
Flow diagram Flow diagram illustrating article identification, screening, eligibility assessment, and selection process. Our systematic literature search yielded 22 studies.

Our systematic literature search focused on the effects of neuromodulation on the CNS in DPN and identified 357 manuscripts. Four additional studies were identified by reviewing reference lists. Of the 361 total manuscripts, 22 studies met the inclusion criteria. Table 1 highlights the biomarkers and physiological pathways modulated by SCS in diabetic neuropathy models and clinical cohorts. The reviewed evidence consistently supports the multimodal effects of SCS on neuroimmune, vascular, and neuronal dysfunction mechanisms underlying DPN pathology (Figure 1).

**Table 1:** Studies reporting stimulation and measured outcomes in DPN These 22 studies met the inclusion criteria and assessed changes in biomarkers and physiological outcomes of SCS in diabetic neuropathy models and clinical cohorts. BMI: body mass index, CCM: corneal confocal microscopy (nerve fiber density), CGRP: calcitonin gene-related peptide, CSA: cross-sectional area, EQ-5D-5L: EuroQol five dimension, HbA1c: hemoglobin A1c, HRQoL: health-related quality of life, IENFD: intraepidermal nerve fiber density, NCV: nerve conduction velocity, NT-3: neurotrophin-3, PGIC: patient’s global impression of change, QSART: quantitative sudomotor axon reflex test, WDR: wide-dynamic-range

Author	Species	Stimulation type	Sample number	Measured outcome
Petersen et al. 2025 [16]	Humans	Epidural	57	HRQoL, EQ-5D-5L, PGIC, HbA1c, and BMI
Han and Cong 2024 [18]	Humans	SCS and DRGS	106	NCV
Zhou and Bao 2023 [20]	Humans	Epidural	19	NCV and revascularization
Koetsier et al. 2023 [42]	Humans	DRGS	9	IENFD
Beauchene et al. 2023 [44]	Rats	PNS	8	WDR
de Geus et al. 2024 [45]	Rats	Epidural	32	IENFD and protein expression
Xu et al. 2023 [46]	Humans	Epidural	46	NCV and revascularization
de Geus et al. 2024 [47]	Rats	Con- and DTM-SCS	28	Spinal lipid expression
de Geus et al. 2023 [48]	Rats	Epidural	64	inflammatory pathways
Wang et al. 2024 [49]	Rats	Epidural	11	IENFD
Chen et al. 2023 [50]	Humans	Epidural	6	CCM, IENFD
Ni et al. 2023 [51]	Rats	Epidural	6	Inflammatory markers
Liu et al. 2025 [52]	Humans and rats	Epidural	10 humans and 5 rats	NT-3 and revascularization
Yakhnitsa et al. 1999 [53]	Rats	Epidural	61	WDR
Lin et al. 2014 [54]	Rats	PNS	10	NCV, CGRP, and revascularization
Kissoon et al. 2023 [55]	Humans	Epidural	9	QSART, NCV, and revascularization
de Vos et al. 2009 [56]	Humans	Epidural	9	Microcirculation
Zhou et al. 2024 [57]	Humans	Epidural	102	Revascularization
Liu et al. 2024 [58]	Humans	Epidural	36	Foot circulation
Petrakis et al. 2000 [59]	Humans	Epidural	60	Revascularization
Canós-Verdecho et al. 2025 [60]	Humans	Epidural	19	IENFD
Amorizzo et al. 2023 [61]	Humans	Epidural	1	CSA of peripheral nerves

Effects of neuromodulation on the CNS in DPN

Over the years, our understanding of SCS has evolved from a purely analgesic intervention into a therapy with the potential to reverse key pathological features of PDN [14,45,46]. Specifically, SCS appears to recalibrate neuroimmune responses, restore neuronal integrity, and improve autonomic and vascular function, making it a compelling candidate for disease modification rather than symptom suppression alone [45,62,63]. These effects lead to measurable improvements in overall neurological function, including sensation, which may help lower the risk of foot ulceration and falls [64].

SCS seems to effectively recalibrate the immune system within the spinal cord by managing microglia and inflammatory pathways. For example, SCS has been shown to reduce local inflammation and pro-inflammatory lipid levels in the dorsal horn of the spinal cord [47]. Moreover, in rodent models of nerve injury, stimulation reduces microglial activation by lowering colony-stimulating factor 1 (CSF1) levels [62], lessening neuropathic pain. Other paradigms, such as high-frequency and differential-target multiplexed SCS, further tip the balance toward anti-inflammatory signaling and dampen pain behaviors in the PDN [48,63].

Over sustained treatment periods, conventional SCS may promote nerve repair. Animal studies report increased intraepidermal nerve fiber density (IENFD) and lower levels of pro-brain-derived neurotrophic factor in the spinal cord, suggesting that SCS can restore both the structure and function of pain pathways [45]. In contrast, burst SCS, which delivers shorter, intermittent stimulations, achieves comparable analgesic effects by modulating central pain processing and suppressing aberrant neuronal firing, without inducing paresthesia. However, it may not consistently promote new fiber growth [49,50]. Mechanistically, SCS has been shown to suppress neuropathy-induced TLR4 (toll-like receptor 4) and NFκB (nuclear factor-kappa B) p65 (phosphorylated subunit 65) activation, thereby reducing pro-inflammatory, pain-promoting cytokines such as IL-1β (interleukin), IL-6, and TNF-α (tumor necrosis factor alpha) in the spinal dorsal horn [51]. SCS has been shown to induce neurotrophin-3, which may contribute to nerve regeneration in diabetic feet [52]. Taken together, SCS not only relieves discomfort but also addresses the underlying maladaptive changes of DPN in the neuronal and molecular components of the spinal cord.

Recent evidence suggests that SCS may modulate local vascular transcription factors within the spinal cord, thereby promoting improved microcirculation, as with its established macrovascular effects in peripheral and cerebral circulation, and this correlates with its analgesic benefits [46]. It remains to be determined whether SCS can similarly restore spinal cord microcirculation by modulating local vascular transcription factors and how this would contribute to pain relief in DPN [46,65,66].

Importantly, SCS also rebalances higher-order circuits. Animal studies demonstrate that SCS can engage both segmental and brain-level pathways, recruiting inhibitory neurotransmitters, including GABA, serotonin, and norepinephrine, to produce widespread pain relief [67,68]. SCS has been shown to counteract this overexcitability by reducing glutamate and aspartate release through local GABAergic mechanisms [69] and suppressing both evoked and spontaneous discharges in WDR neurons, thereby restoring spinal signaling balance and alleviating neuropathic pain [53].

The intricate balance of the autonomic and enteric nervous systems that govern digestion can also be disrupted by DPN. As a result, many patients experience slowed gastric emptying (gastroparesis) and sluggish small-bowel transit, leading to symptoms like nausea, bloating, and abdominal discomfort [70]. Evidence suggests that SCS may help restore gastrointestinal function by dampening overactive sympathetic signals and reestablishing a healthier autonomic balance. Preliminary clinical studies report improvements in abdominal pain and related symptoms in patients with gastroparesis treated with SCS. However, larger, controlled studies are still needed to confirm its effects on measurable motility parameters, such as gastric emptying times [71,72].

While the precise mechanisms by which DRGS alleviates pain remain under investigation, early evidence suggests it may share certain anti-inflammatory and metabolic effects with SCS. By dampening local inflammation and rebalancing metabolic stress within the ganglion, targeted DRGS may help interrupt the feed-forward cycle of hyperexcitability that drives neuropathic pain in DPN [18,62]. Notably, unlike conventional SCS, DRGS does not appear to induce GABA release from spinal dorsal horn neurons, and contrary to earlier assumptions, it also does not modulate GABAergic signaling within the DRG itself [73-75]. This indicates that DRGS likely operates through distinct, non-GABAergic pathways to modulate nociceptive transmission. Clinically, DRGS is already approved for several chronic neuropathic pain conditions, including complex regional pain syndrome and chronic postsurgical pain, and is gaining interest for nonsurgical back pain and peripheral neuropathies [76]. As research progresses, DRGS may offer a more precise, anatomically targeted approach for patients whose pain arises from segmental or localized nerve dysfunction, such as diabetic neuropathy.

**Figure 2:**
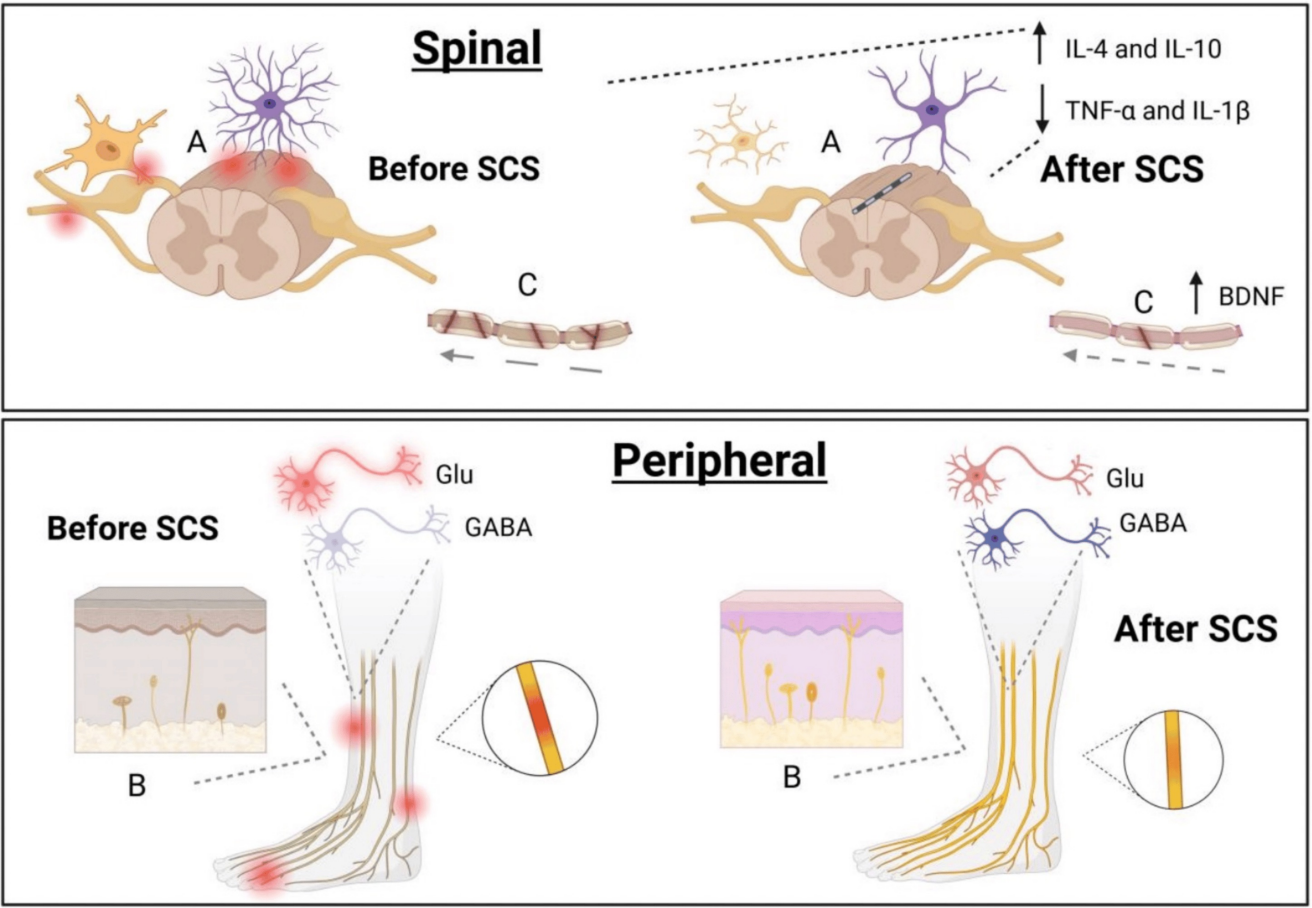
Therapeutic effects of SCS on improving the structural and functional deficits of DPN DPN and PDN cause changes in the spinal (top) and peripheral (bottom) nervous systems that SCS may partially recover. BDNF: brain-derived neurotrophic factor, Glu: glutamate, IL: interleukin, TNF: tumor necrosis factor, WDR: wide-dynamic-range Image Credit: Authors. Created using BioRender.

Preclinical studies using diabetic neuropathy models have demonstrated that a single, one-hour pulse of PNS to the sciatic nerve after injury can jump-start regeneration [54,77]. Treated animals exhibited upregulation of growth-associated genes in their dorsal root ganglia, β-tubulin, GAP-43, and Sonic hedgehog, compared to unstimulated controls [77]. Furthermore, evidence suggests that PNS can normalize calcitonin gene-related peptide expression in the lamina I-II regions of the dorsal horn in animal models [54]. As these techniques mature, head-to-head comparisons of PNS versus SCS, along with their underlying molecular effects, will be essential for clarifying which modality best benefits each patient (Figure 2, Table 2).

**Table 2:** Pathophysiological changes in DPN and the therapeutic effects of SCS This table summarizes key structural, functional, and biomarker alterations associated with DPN and highlights how SCS modulates these abnormalities. Improvements include enhanced nerve regeneration, reduced neuroinflammation, and partial restoration of neural and vascular functions. NS: not significant, PAG: periaqueductal gray, VEGF-A: vascular endothelial growth factor-A, IENFD: intraepidermal nerve fiber density, LDF: laser Doppler flowmetry, DPN: diabetic peripheral neuropathy, SCS: spinal cord stimulation

Parameter	Observed in DPN	Effect of SCS	References
IENFD	Reduced small fiber density	Increased at 6–12 months	Chen et al. 2023 [50]; Canós-Verdecho et al. 2025 [60]
Corneal nerve fiber density	Decreased density and branching	Significantly increased	Chen et al. 2023 [50]
Nerve conduction velocity	Slowed conduction, increased latency	Improved post-SCS	Han and Cong 2024 [18]; Zhou and Bao 2023 [20]
Synaptic plasticity	Increased dorsal horn maladaptive plasticity and information	Normalized by SCS	Lin et al. 2017 [30]; de Geus et al. 2024 [47]; de Geus et al. 2023 [48]
Spinal inflammation	Elevated neuroinflammation	Suppressed by SCS	Wang et al. 2024 [49]; Ni et al. 2023 [51]
Supraspinal modulation	Altered connectivity and thinning	Restored by SCS (PAG, thalamus)	Segerdahl et al. 2018 [39]; Chao et al. 2022 [40]
LDF	Impaired skin perfusion	Trends toward improvement (NS)	Kissoon et al. 2023 [55]; de Vos et al. 2009 [56]
Doppler ultrasound	Reduced large vessel flow	Revascularization at 6 months	Xu et al. 2023 [46]; Zhou et al. 2024 [57]
VEGF-A expression	Decrease in spinal cord	Increase with SCS	Ved et al. 2018 [21]; Lobo et al. 2022 [36]

Overall, SCS shows significant promise in mitigating the maladaptive changes of DPN by modulating inflammation, enhancing neuronal repair, and restoring function. Further research is needed to optimize these therapies and elucidate their effects on microcirculation and autonomic dysfunction, thereby paving the way for more effective, targeted treatments for patients with DPN. Determining the direct effects of neuromodulation on key biomarkers of DPN in the CNS is a critical foundation for this future research.

Discussion

Various diagnostic tools have identified biomarkers in the peripheral and central nervous systems that aid in the diagnosis and staging of DPN. These neurophysiological biomarkers may also be used to evaluate the efficacy of SCS and related neuromodulation strategies to reverse the structural and functional deficits of DPN. Electrophysiological testing, including nerve conduction studies, remains the clinical benchmark for confirming DPN and detecting abnormalities in approximately 80% of peroneal nerves and 83% of sural nerves [78]. Importantly, slower conduction velocities not only reflect nerve injury but also herald a higher risk of foot ulcers and even amputation [79]. Notably, several clinical series have reported that SCS can partially reverse these electrical deficits, thereby improving conduction velocities in both motor and sensory fibers [18,20]. While the precise drivers of this recovery are still under investigation, it involves a combination of enhanced microvascular perfusion and direct modulation of spinal circuits that govern nerve excitability. Indeed, some evidence suggests that microcirculatory breakdown may precede and precipitate the drop in conduction velocity [80], whereas other studies argue that primary nerve injury itself contributes to vascular dysregulation [81]. Unraveling how SCS helps restore vascular health in the diabetic foot and, in turn, supports nerve function remains a vital area for future research.

Laser Doppler flowmetry (LDF) provides a noninvasive measure of the skin’s microcirculation by illuminating the skin with a low-power laser and measuring the Doppler shift caused by moving red blood cells [82]. In diabetic neuropathy, this method often reveals blunted blood-flow responses, reflecting the microvascular damage that accompanies nerve injury. Although SCS has been celebrated for its neural benefits, its impact on skin microcirculation appears modest; studies report only minor changes in LDF parameters, although longer treatment courses suggest subtle improvements [55,56]. By contrast, Doppler ultrasound of larger vessels demonstrates clear revascularization and better perfusion after six months of SCS [46,57]. Other studies reported an increase in foot temperature, a measure of foot circulation, as early as two weeks after surgery [58]. Taken together, these findings suggest that while SCS can meaningfully enhance macrovascular flow, fully restoring microvascular health in the skin may require additional or prolonged interventions. Earlier studies, however, suggest that the stage of neuropathy influences the success rate of SCS in improving blood flow [59]. A large multicenter randomized controlled trial is currently investigating the effect of SCS on microcircuitry in patients with DPN, which could reveal the systemic impact of this intervention [83].

Although the longest axons are particularly susceptible to diabetic nerve injury, the cornea has emerged as an equally sensitive indicator of small-fiber damage [84]. Corneal confocal microscopy (CCM) provides a window into this process, capturing high-resolution images of the sub-basal nerve plexus. This enables noninvasive measurement of nerve fiber density, length, and branching, three metrics that decline in parallel with neuropathy severity [84]. Notably, six months after spinal cord stimulator implantation, patients with PDN exhibit a significant rebound in corneal nerve density, suggesting that SCS may promote small-fiber regeneration well beyond its effects in the lower limbs [50].

Furthermore, one of the earliest and most reliable biomarkers of diabetic neuropathy is a drop in IENFD. Clinically, this is assessed by obtaining a small skin biopsy, typically from the distal leg, and using immunohistochemical stains to quantify the nerve fibers in the epidermis. Because these small fibers are among the first to suffer from diabetes, their loss closely tracks pain symptoms [84,85]. Excitingly, SCS does more than ease discomfort; it can help regenerate these fibers. In patients with PDN, IENFD rises significantly by six months after SCS implantation and continues to improve through one year [50,60]. By contrast, DRGS, while effective at reducing pain, has not shown meaningful IENFD changes after two years of treatment [42]. These findings suggest that SCS may offer a unique capacity to restore cutaneous nerve integrity, potentially translating into more lasting relief for patients.

Tibial nerve ultrasound is gaining traction as an additional painless imaging modality for detecting structural alterations in PDN. A larger cross-sectional area of the tibial nerve is associated with more severe neuropathic changes, suggesting it may serve as a reliable imaging marker of disease burden [86]. A case report has shown a reduction in the cross-sectional area of the tibial nerve in response to SCS treatment [61].

The quantitative sudomotor axon reflex test (QSART) provides a sensitive measure of autonomic small-fiber integrity by quantifying sweat output in response to acetylcholine iontophoresis, thereby directly probing postganglionic sympathetic axons [87]. In diabetic neuropathy, QSART often uncovers early sudomotor deficits, sometimes before patients notice any symptoms, making it a valuable tool for detecting autonomic involvement. Notably, although SCS reliably alleviates pain and promotes regeneration of sensory fibers, QSART assessments indicate that autonomic dysfunction can persist despite effective neuromodulation [55]. This dissociation suggests that although SCS favorably affects many neural pathways, it may fall short of halting the broader course of autonomic fiber degeneration. Consequently, a comprehensive management plan for diabetic neuropathy should integrate treatments targeting both somatic and autonomic components to fully address the multifaceted nature of the disease.

Magnetic resonance neurography (MRN) is a high-resolution technique for visualizing peripheral nerve pathology in PDN, revealing nerve thickening and T2-weighted hyperintense lesions that correlate with symptom severity; patients with PDN show greater lesion load and distribution than those with painless DPN or no neuropathy [88]. Shear-wave elastography similarly detects increased nerve stiffness in DPN compared with healthy controls [89]. The Hoffman reflex rate-dependent depression (HRDD) assesses spinal inhibition, which is blunted in painful and exaggerated in painless DPN, suggesting distinct pain-dependent neurophysiological pathways [90]. Although SCS modulates spinal and supraspinal circuits, its effects on MRN, nerve elasticity, or HRDD remain unexplored. Prospective studies combining MRN, tibial nerve ultrasound, and HRDD before and after SCS could reveal structural and functional changes in peripheral nerves, highlighting these markers as key outcomes for understanding SCS mechanisms in diabetic neuropathy.

Recently, noninvasive transcutaneous SCS has been shown to reduce pain intensity and alter spinal excitability in conditions such as phantom limb pain, suggesting potential applicability to PDN treatment and warranting further investigation [91]. However, limitations remain regarding the extent of autonomic innervation influenced by SCS. Future studies should investigate optimal stimulation parameters, whether sensory improvements result from regeneration or refunctional sensory processing, and how SCS modulates neuroinflammation and glial cells. Furthermore, despite advances, the exact mechanisms underlying SCS modulation of autonomic and sensory pathways in patients with diabetic neuropathy remain understudied.

## Conclusions

Diabetic neuropathic pain emerges from a quagmire of metabolic disturbances, nerve fiber injury, small-vessel damage, and chronic inflammation that spans the entire sensory pathway. Although conventional medications can ease discomfort for some, many individuals still endure stubborn, drug-refractory pain. SCS has gained momentum as an alternative treatment strategy that effectively blocks pain, modulates inflammatory cascades, promotes neural repair, and improves glucose regulation. Specifically, SCS reduces proinflammatory biomarkers, such as CSF1, and proinflammatory lipids in the dorsal horn of the spinal cord while promoting IENFD and decreasing pro-brain-derived neurotrophic factor levels, thereby addressing the molecular underpinnings of DPN. Furthermore, different SCS modalities, such as conventional, high-frequency, and differential target-multiplexed stimulation, have been shown to modulate distinct aspects of the inflammatory pathways involved in DPN.

Although SCS has opened exciting avenues in DPN treatment, we still lack a clear picture of its long-term impact on key processes, including neuroinflammation, blood-nerve barrier integrity, and pain processing above the spinal level. Pinpointing exactly how SCS influences both structural and functional biomarkers is critical for fine-tuning these therapies to maximize individual patient benefit. At the same time, more focused modalities, such as DRGS and PNS, are enabling personalized, region-specific relief that aligns more closely with each patient’s unique pattern of nerve injury and pain. Equally important is the development of clearer selection criteria and personalized treatment strategies to ensure that everyone receives the neuromodulation modality best suited to their unique profile. Ultimately, combining SCS with targeted metabolic management and rehabilitation could foster a truly multidisciplinary paradigm for tackling DPN and PDN, one that not only eases pain but also addresses the diverse contributors to this complex, life-altering condition.
